# Adding Mealworm (*Tenebrio molitor* L.) Powder to Wheat Bread: Effects on Physicochemical, Sensory and Microbiological Qualities of the End-Product

**DOI:** 10.3390/molecules27196155

**Published:** 2022-09-20

**Authors:** Magdalena Gantner, Katarzyna Król, Anna Piotrowska, Barbara Sionek, Anna Sadowska, Klaudia Kulik, Mateusz Wiącek

**Affiliations:** 1 Department of Functional and Organic Food, Institute of Human Nutrition Sciences, Warsaw University of Life Sciences, Nowoursynowska Str. 159c, 02-776 Warsaw, Poland; 2 Department of Food Gastronomy and Food Hygiene, Institute of Human Nutrition Sciences, Warsaw University of Life Sciences, Nowoursynowska Str. 159c, 02-776 Warsaw, Poland

**Keywords:** edible insects, mealworm, novel protein, entomophagy

## Abstract

Entomophagy, that is, the consumption of insects, is gaining more and more popularity. The research carried out so far on the use of edible insects in the food industry has shown that they are a valuable source of protein, and do not significantly affect the functional and sensory properties of food. Edible insects also contribute to sustainable, environment friendly food production. Taking the above into account, the influence of adding insect powder on the physicochemical properties, sensory characteristics, and microbiological qualities of wheat bread was evaluated. This study aimed to partially replace wheat flour (5, 10, and 15%) in bread with mealworm powder (*T. molitor*) to produce protein-fortified bread. Bread containing mealworm powder showed similar density and water activity compared to the control wheat bread. The addition of mealworm powder did not negatively affect the properties of bread. The total color difference increased significantly (*p* < 0.05) with the insect flour share in bread formulation and ranged between 2.27 for M5, 4.00 for M10, and 4.50 for M15. The protein content in bread fortified with 5–15% mealworm powder increased by 15–59% compared to the control bread, whereas fat content increased by 35% to 113%. Results of sensory evaluation revealed that modification of the recipe, depending on the mealworm powder addition level, significantly (*p* < 0.05) affected bread color, odor, flavor, and overall sensory quality. The research showed that the optimal enrichment level is using 5% mealworm flour in the bread recipe. Moreover, the obtained variants of bread were characterized by good microbiological quality after baking. In bread M10, no yeasts and molds were found during a period of 2 days of storage. The number of yeasts and molds in the other bread variants was relatively low. To conclude, the results confirmed the usefulness of insect powder in making protein-fortified bread of good quality comparable to traditional wheat bread.

## 1. Introduction

In recent years, insects have gained more attention in Europe as a sustainable source of protein. Since 2003, the Food and Agriculture Organization (FAO) recognized the potential of using edible insects for food and feed. This supports several topics related to edible insects, including knowledge generation and sharing through publications and awareness raising on the role of insects in many countries worldwide [1]. More significantly, the new regulation established by the European Union (EU) 2021/882 authorizes the placing on the market of dried *T. molitor* larva as a novel food [2]. Therefore, they will become the first edible insects in the European Union. The changes in the regulation are essential because food and feed sources are needed for the continually growing world population and can contribute to global food security. Moreover, the increasing demand for protein-rich food (especially meat) and livestock feed contributes to climate change [3,4,5]. 

Edible insects are a good source of essential amino acids, polyunsaturated fatty acids, certain vitamins, and minerals [6]. Protein content varies among and within insect orders from 13 to 77% [7,8] with protein digestibility of 76–98% lower than animal proteins, but higher than many plant proteins [9]. The fat content of edible insects varies between 10–50% [10].

The major challenge for the food industry is making edible insects more acceptable for consumers. Insects can be consumed at different stages of their development, such as eggs, larvae, pupae, and adults, but most of the registered species are consumed in the form of larvae or pupa. In general, Western society may be reluctant to accept insects as a protein source because they have never played an essential role in their food culture [11]. One of the methods to increase the acceptance of insect-based food products is to change the presented form. The study presented by Schösler et al. [12], showed that the consumer acceptance of insects in food products increased when insects were not visible in modified products indistinguishable from familiar ones. As is known, visual impressions are crucial because they are the consumer’s first chance to form an opinion of the product. Whole insects are not popular in Western societies; therefore, it is favorable to introduce insects to the human consumer in a masked form such as powder, whole meal, or fraction [13,14]. 

Recently, there has been a growing number of papers discussing the use of insects in common food products, and some reformulations have been described, [15,16,17,18,19]. Edible insect can be consumed whole or after being processed into flour, powder, pastes or as extracted protein [20]. Therefore, bakery products, such as breads, biscuits or snacks are most often supplemented with edible insects (*Acheta domesticus* L., *Alphitobius diaperinus P*., *T*. *molitor* L.), and affect not only the nutritional value, but also the technological parameters and sensory properties in final product [15,21,22,23,24]. Edible insects are also used in other food products. Kim et al. (2016) [25] confirmed the positive effect of the addition of mealworm larvae and silkworm pupae meals (10%) on the properties of emulsion sausages, e.g., increasing the cooking yield. Moreover, the larval mealworm (*T.molitor)* can be used as a meat substitute in hamburger formulations or for obtaining oils [26,27]. According to Zielińska and Pankiewicz [15] cereal-based foods such as bread, biscuits, and bakery products are prevalent and highly accepted worldwide. Thus, research on how to enrich them with insect powder would be a good starting point. Considering that edible insects such as mealworm (*T. molitor*), available as a finely ground flour-like powder, are a source of nutrients, this work investigated the effect of partially replace wheat flour in bread with mealworms powder (*T. molitor*). The study investigated the impact of some flour with insect powder could influence bread’s sensory, physicochemical and microbiological properties. The used substitution was to demonstrate the potential use of mealworm powder in food technology and at the same time to obtain bread enriched with nutrients from mealworm (mainly wholesome protein).

## 2. Results and Discussion

### 2.1. Physicochemical Properties of Mealworm Powder and Wheat Flour 

**Figure 1:**
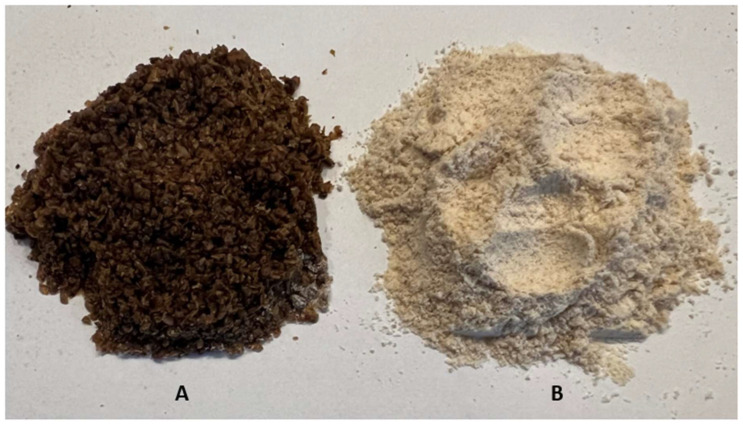
Pictures of the mealworm powder (**A**) and wheat flour (**B**) used for bread making.

Table 1 shows some physicochemical properties of mealworm powder and wheat flour. The tested preparations differed statistically significantly (*p* < 0.05) in pH, color coordinates, and water activity, while they were similar in water and fat absorption. Wheat flour was characterized by a lower pH, amounting to approx. 6.78, compared to the mealworm powder. The water activity was characterized by the higher value, amounting to approx. 0.44, when compared to powder, where it was 0.33. The degree of water and fat absorption was similar for both preparations; it was 1.47–1.63 g water/g powder and 0.26–0.40 g fat/of g powder, respectively. As in the present study, in the work of Roncolini et al., 2019 [27], no significant differences were noticed in the percentage of water absorption for wheat flour and the tested mixtures of wheat flour with mealworm powder at two levels of 5 and 10% substitution. The L* value of flour and powder varied from 32.65 to 80.48, respectively. The other color coordinates, a* and b*, also significantly differed and ranged between 4.73–5.84 and 21.54–25.31, respectively. Moreover, as is seen in Figure 1, wheat flour was characterized by high fragmentation of particles in contrast to the mealworm, where the elements of the chitin were easily visible. 

### 2.2. Designing of the Mealworm Bread 

Three different amounts of mealworm powder were used in addition to traditional wheat bread to produce experimental bread. In Table 2, the formulations of doughs obtained using the different ratios of wheat flour and mealworm powder are presented. The mealworm powder was used to substitute wheat flour content of 5, 10, and 15% (*w*/*w*). Substitution of wheat flour with mealworm powder did not significantly affect the characteristics of the raw dough. The obtained bread doughs did not differ from one another in the homogeneity of the samples. Dough with the addition of mealworm powder showed higher viscosity (organoleptically assessed); the higher the powder addition, the higher the viscosity. Nevertheless, it did not cause great difficulties in forming the loaves. In the studies of Roncolini et al., (2019) [27], the possibility of adding mealworm (*Tenebrio molitor* L.) powder to bread dough was investigated at the level of substitution of 5% and 10% of wheat flour. In that study, the addition of powdered mealworm did not adversely affect the features of both the raw dough and the finished bread. All the trials made showed the same acidifying capacity, and the bread containing 5% addition of mealworm powder showed the highest volume and the lowest hardness. The addition of mealworm powder negatively influenced the instrumentally determined dough strength; the greater the amount of added preparation, the lower the strength obtained, which was probably due to the reduced amount of total gluten in the dough [27].

### 2.3. Assessment of Quality Parameters of Designed Mealworm Bread

**Figure 2:**
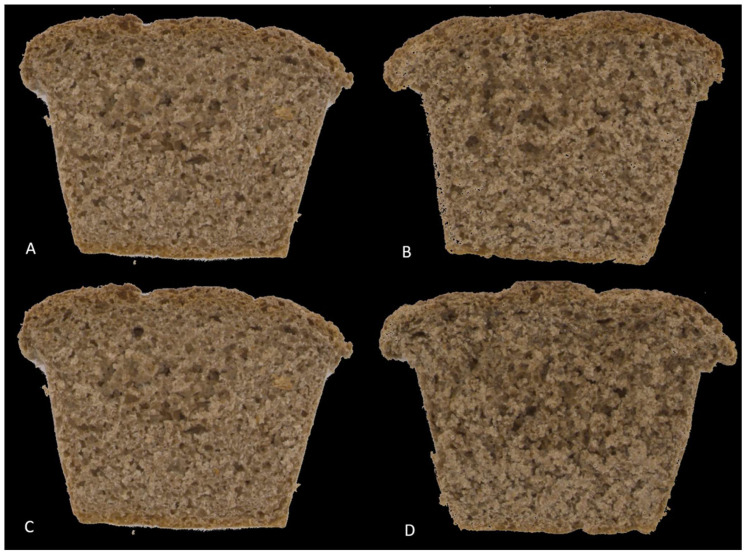
Picture of bread loaves. (**A**) control sample—100% wheat flour, (**B**) mealworm powder 5%, (**C**) mealworm powder 10%, (**D**) mealworm powder 15%.

Edible insects are considered one of the most promising alternative sources of protein to solve the global problem of protein production. Insects also present large amounts of fats and minerals, especially potassium, calcium, iron, and magnesium [6,28]. Furthermore, the main advantage of insects over other sources of protein is the low environmental cost of production, which is essential to satisfy the global protein demand [29]. Moreover, recently published studies have shown a greater willingness to consume products with shredded larvae than whole insects [30,31]. In Figure 2, the experimental sliced bread samples are presented. 

The color, texture, and volume of bakery products influence consumer choice and acceptance. The values of CIE L*a*b* coordinates, the density of bread loaf, water activity, and hardness are presented in Table 3. The tested preparations differed statistically significantly (*p* < 0.05) in L*, a*, b*, browning index, and hardness.

The lightness (L*) values of the bread ranged between 44.87 for control and 40.07 for bread with 15% of *T. molitor* addition; significant differences (*p* ≤ 0.05) were observed between crumbs of bread. The darker color of bread in both M5, M10, and M15 addition of insect powder was attributed to the higher protein content compared to control. These results were confirmed by studies by Khuenpet et al. [31] and Cabuk [32] on bread and muffins produced with mealworm powder addition. Additionally, the formulation with insect powder decreased the redness tonality (a*) significantly only in bread with 15% of *T. molitor* addition compared to the control. The results obtained by Garcia-Segovia et al. [33] and Cabuk [32] were different, and showed an increased value of a* parameter by mealworm (*T. molitor*) powders in bread and muffins. Most likely, this was caused by the color of the bread flour and its dark tonality. The yellow color indicated by b* value was significantly reduced for M10 and M15 compared to the control. This trend for change might be attributed to the color of insect powder which was confirmed by Cabuk’s [32] research. 

The best way to interpret L*a*b* values is to determine the total color difference (ΔE) and browning index (BI). The total color difference increased significantly with the increase of insect powder share in the bread formulation. Previous studies pointed out that color difference is visually noticeable for consumers when ΔE ˃ 3.0 [34]. In our study, the total color difference values of the bread ranged between 2,27 for M5, 4,00 for M10, and 4,50 for M15. This result proves that the M10 and M15 breads were darker in color both in instrumental tests and were noticeable to consumers, which may translate into lower acceptability due to significant colors compared to traditional wheat breads.

Bread with mealworm powder showed a significantly higher BI value than the control, and it can be assumed that the share of the raw material with a higher protein content contributed to the intensity of the Maillard reaction, which takes place during the baking process. This was also confirmed by Gonzales et al. [22] and Kowalski et al. [35], who researched the bread with various edible insect flour fortifications. 

According to the obtained results, no significant differences in terms of water activity were observed among the analyzed samples and was 0.96–0.97. The density ranged between 0.36 ± 0.00 g/cm^3^ for 10% insect powder addition and 0.43 ± 0.02 g/cm^3^ for control, wheat flour bread. There were no significant differences in this parameter depending on the proportion of mealworm powder and wheat flour. Khuenpet et al. [31] measured specific volume parameter expressed as a ratio of loaf volume (cm^3^) to loaf weight (g) and observed a decrease in the parameter value with an increase share of insect powder. In the case of the control sample (only wheat flour bread), the highest value of specific volume was determined. This could be related to the type of wheat flour used and may be caused by the weakening of the gluten strength by adding another flour of a different origin [22]. Thus, it is necessary to take into account the effect of such an addition, as a gluten-free factor may reduce the baking value.

**Figure 3:**
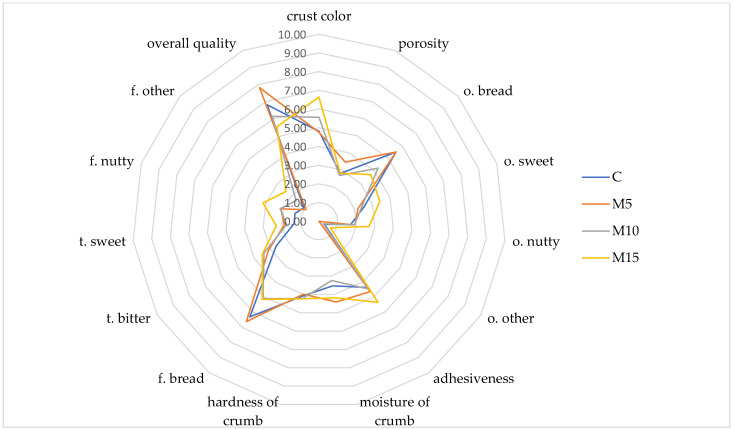
Sensory score plot of the breads with addition of mealworm powder (f.—flavor, t.—taste, o.—odor).

A significant effect on the increase in the hardness of slices was observed only with a 15% share of mealworm powder. The breads with 5% and 10% addition of mealworm powder did not differ significantly in this parameter compared to control bread (wheat flour). González et al. [22] did not observe any significant effect of mealworm flour on hardness compared to control wheat bread. Moreover, Roncolini et al. [27] observed that 5% and 10% addition of mealworm flour caused a decrease in bread loaf slices hardness, probably due to its lipid fraction. The sensory analysis results also confirmed no differences in the hardness of crumbs between bread with the addition of mealworm powder and wheat flour bread (Figure 3).

As expected, reformulating wheat bread with mealworm increased protein and fat (Table 4). More specifically, protein content in bread fortified with 5–15% mealworm powder increased by 15–59% compared to the control bread, whereas fat content increased by 35% to 113%. These data are in close agreement with results obtained by Oliveira et al. [30], Gonzalez et al. [22], Roncolini et al. [27] for bread enriched with insect powders. According to the literature, edible insects, apart from the high content of wholesome protein, are characterized by a high content of B vitamins and minerals. Among the minerals, iron and zinc have the largest share [36]. Copper, manganese, magnesium and calcium are also present in smaller amounts [37]. Preparations from edible insects are also a source of thiamine (0.1–4.0 mg per 100 g of dry weight), riboflavin (0.1–8.9 mg), cobalamin (0.5–8.7 μg per 100 g), also folic acid and, in smaller amounts, retinol and β-carotene) [37,38,39]. 

Sensory evaluation showed that the body of the tested bread was characterized by a light brown color, the intensity of which increased with a higher mealworm powder addition (Figure 3). The body of the bread with the addition of mealworm at 15% was statistically significantly darker than the control sample. 

No significant influence of changes in the bread recipe on pore size was found. In terms of the odor attributes, the bread odor dominated in all the samples, the intensity of which decreased with the higher level of mealworm flour addition—in the M10 and M15 samples, the bread smell intensity was statistically significantly lower compared to the control sample (C) and M5. The bread also had a noticeable sweet and nutty smell of significantly higher intensity in the M15 test. The examined bread did not differ significantly regarding texture attributes assessed in the oral cavity. The body of the bread was quite soft, moderately moist, and adhesive. 

Concerning the flavor descriptors, it was found that the higher addition of mealworms (10% and 15%) significantly reduced the intensity of the bread flavor. Recipe modification also influenced the intensity of bitter taste and nutty flavor—bread with the highest mealworm flour addition (15%) was characterized by a significantly higher intensity of these attributes compared to the control sample.

The conducted research showed that the optimal enrichment level is using 5% of mealworm flour in the bread recipe. The overall sensory quality of this bread, understood as an appropriate harmonization of all sensory attributes, was the highest—it did not differ statistically significantly from the control sample. At the same time, it was statistically significantly higher than the breads with a higher addition of mealworm powder (10% and 15%).

The results of our research are in line with the literature data which show that edible insect powders are useful in the production of bakery goods. Their use improves nutritional value and affects the product’s properties including sensory attributes. The quality of the enriched products depends, inter alia, on the insect species, method of powder production and the bakery product recipe; therefore, the level of the enrichment must not be too high and must be determined in studies [22,40]. Similar to our studies, the results of the research by Haber et al. (2019) [40] indicate a significant effect of the insect powder adding on the smell of bakery products—grasshopper (*Schistocerca gregaria*) powder adding at the level 200 g/kg negatively affected wheat bread sensory quality, mainly due to its distinctive odor, which was not accepted by consumers, whereas the odor liking scores of the bread enriched at the level 100 g/kg was comparable to the control sample. Literature data also indicate a significant effect of the insect powder addition on the color of bakery products, which was also shown in our research. The color of wheat bakery products turns brownish with increasing levels of insect powder addition [22]. However, this does not negatively affect the willingness to eat. Research conducted by Haber et al. (2019) [40] has shown that the differences in color intensity did not affect the consumers’ acceptance of the bread enriched with insect powder. The color preference was similar between the enriched and the control samples despite the clear difference in bread color. Regarding texture, our research did not show any significant effect of mealworm powder addition on texture attributes assessed in the oral cavity. In the study of Mafu et al. (2022) [41], whole wheat bread enriched with the addition of cricket powder at the 20% level had a hard texture and required a lot of chewing force compared to the control samples, but at the same time it was characterized by the best consumer acceptance. In turn Haber et al. (2019) [40] showed that the texture-liking scores for bread enriched with grasshopper powder, regardless of the addition level (100 g/kg and 200 g/kg), were comparable to the control bread with a slight increase in the sample with the highest level of insect powder enrichment, which was characterized by softer texture.

### 2.4. Microbiological Analysis 

Bread belongs to easy spoilage products, and adverse changes begin to appear in it immediately after baking. These undesirable processes are associated with moisture loss, the staling process, and the development of bacteria, molds, and yeasts [42,43,44]. The way to extend the shelf life of bread is by microbial fermentation or using natural preservatives such as plant extracts and essential oils [19,20,21,22,23,24,25,26,27,28,29,30,31,32,33,34,35,36,37,38,39,40,41,42,43,44,45,46,47,48,49]. Bread durability can be extended with preservative use; however, consumers often do not approve of their use [50]. 

Total viable counts (TVC) and yeasts and molds of the bread are given in Table 5. The obtained variants of bread were characterized by good microbiological quality after baking. Total viable counts in all tested breads were low: from 3.51 to 4.22 log CFU g^−1^ for the control and M15 bread, respectively. There was no presence of yeasts and molds in any tested bread. The total viable counts of bacteria, yeasts, and molds may be an indicator of estimate the shelf life of storage bread [51,52]. There were no differences (*p* < 0.05) in the total viable counts between breads with the addition of insect powder. On the second day of storage, there was an increase in TVC of about 2–3 logarithmic orders in the studied breads. Sagar and Pareek [51] also observed an increase in total viable bacteria counts in multigrain bread (to 104 CFU g^−1^) during storage at 28 °C and 70% relative humidity. Ravimannan et. al. (2016) [53] found an increase in total viable counts in white bread during five days of storage at 29–31 °C, which, according to the authors, was caused by improper processing, storage, and the absence of preservatives. In the studied bread M10, no yeasts and molds were found during 2 days of storage. The number of yeasts and molds in the other bread variants was relatively low during 7 days of storage. 

## 3. Materials and Methods 

### 3.1. Nutritional Value, Physicochemical Properties of Wheat Flour and Mealworm Powder

Bread wheat flour type 1850 was purchased on the local market (Melvit, Poland). Mealworm (*T.* *molitor*) powder was produced by ZRIP insects (Wien, Austria). According to the information on the label, insects were roasted before griding to enhance the palatability of the powder. In Table 6, detailed mealworm powder and wheat flour characterization were presented.

pH value was measured using the potentiometric method with a handheld pH meter (Model 205, Testo AG, Germany). The pH values were measured in a 5% wheat flour and mealworm powder solution. The values were measured in triplicate. To measure the water activity, 5g of wheat flour or mealworm powder was placed in a measuring cup in the device (AquaLab 4TEV, München, Germany), and the measurement was performed in triplicate.

The water holding capacity (WHC) analysis was carried out on 1 g of the protein powder or wheat flour. The sample was homogenized using an ultrasonic homogenizer (Hielscher UP400ST, Berlin, Germany) with 30 mL of distilled water and then centrifuged for 15 min (6000 RPM, 0 °C) in a centrifuge (MPW-380 R, MPW Med. Instruments, Warsaw, Poland). After flooding with complementary water, samples with sediment were left upside down for 10 min and then weighed. The WHC was determined in triplicate for each sample by weighting the samples at the beginning and after the centrifugation of sampling and calculated based on mass difference. The measurement was expressed as a gram of absorbed water per one gram of flour or powder. The fat absorption capacity (FAC) was determined using a similar procedure but rapeseed oil. The FAC was expressed as the amount of oil per one of flour or powder and determined in triplicate.

### 3.2. Bread Preparation 

The bread was prepared in three variants with different amounts of added mealworm flour, partially replacing the wheat flour with 5,10, and 15%. Control bread was prepared without the addition of insect flour. Water, yeasts, salt, and sugar were placed in a mixing bowl and then mixed for 3 min at 37 °C. Then the flour was added, and the dough was mixed for 3 min. The bread dough was put aside for 1 h in a warm place to rise. A table in results section present the complex combination of investigated samples. The bread was obtained through a one-step fermentation process followed by 60 min oven baking at 200 °C (Revolution 4xGN 1/1 999576, Poland). The bread was then allowed to cool at room temperature and subjected to physical and color measurements. Next, the bread was stored in room temperature for one week, to perform additional analyses.

### 3.3. Color, Density and Hardness Measurement 

The color of the bread was measured using the artificial eye Visual Analyzer IRIS (AlphaMos, Tuluse, France). The color measurement was performed in triplicate and bread was randomly selected from the batch. The color was recorded in the CIE L*a*b* scale in terms of lightness (L*) and color (a*—redness; b*—yellowness). Between the reference sample and the test sample the total color difference (ΔE) was calculated using the following equation:

(1) $$\Delta E=\;\;\;\sqrt{{\Delta L}^{2}+{\Delta a}^{2}+{\Delta b}^{2}}\;$$

The browning index was calculated using following eq.:

(2) $$\mathrm{BI}=\frac{100\;\left(x\;-0.31\right)}{0.15},\;$$

where x is determined by

(3) $$x=\frac{a*\;+1.75L}{5.645L*\;+a*\;-0.12b*}$$

The density of bread was determined by pouring into a measuring cup filled with rape seeds. A slice of bread was placed in the center. By observing the increase in the volume of the mixture and knowing the mass of the sample, the density of the tested samples was determined. The measurement was performed with three repetitions for each bread sample. Density of bread was calculated following equation:

Density (g/cm^3^) = loaf weight (g)/loaf volume (cm^3^) (4)

Mechanical features of the crumb, expressed as hardness, was measured on 20 mm thick slices using a texture analyzer (Stable Micro Systems TA.XT2i, USA). The applied settings were: 25 mm diametric aluminum cylindrical probe, 50% deformation, 5 s intermission between the measurements and 5 mm/s probe movement speed. Measurements were done with six repetitions, at room temperature. Three slices from each loaf (from the center) were measured. To measure water activity, 5 g of the bread samples were weighed, then ground and placed in a measuring cup in the device (AquaLab 4TEV, München, Germany), the measurement was performed in triplicate.

### 3.4. Proximate Composition

The proximate composition of bread was carried out in accordance with the Atwater system and parameters such as: protein, fat, and fiber content were calculated based on the nutritional value of products.

### 3.5. Microbiological Analysis

The plate culture technique was used to determine the number of microorganisms. For the enumeration of total number of microorganism Nutrient agar (Merk, Germany) was used according to ISO 4833-1:2013-12/A1:2022-06 [54]. For the determination of the number of yeasts and molds YGC agar (Sabouraud Dextrose with Chloramphenicol LAB-Agar, Biomaxima, Poland) was used according to ISO 21527-1:2008 [55]. The number of microorganisms was expressed as log colony forming units per gram (log CFU g^−1^). 

### 3.6. Sensory Analysis

Sensory characteristics of the bread samples were evaluated using the scaling method according to the ISO standard 4121:2003 [56]. The intensity of each attribute was measured on a linear unstructured scale (0–10 cm). Analysis was performed by trained assessors (six members; women, age rate: 29–55) fulfilling the requirements of ISO standard 8586:2012 [57]. Each sample was analyzed in two independent replications, so the mean values were based on twelve individual results. Individual bread samples of the same size were placed in plastic containers and covered with lids. The samples were differently coded for each assessor and presented in individual random order. Still mineral water was used as neutralizer between samples. Evaluation was performed in the sensory laboratory fulfilling all requirements of ISO standard 8589:-2010 [58]. 

### 3.7. Statistical Analysis

Statistica 13.0 (Tibco Software Inc., Palo Alto, CA, USA) software was used for all statistical processing. One-way analysis of variance (ANOVA) was used for dependent groups with a post hoc analysis of Duncan’s test at a significance level *p* < 0.05. 

## 4. Conclusions

The results of this study demonstrated the possibility of using mealworm powder in baked products. Obtained results showed that it is an adequate ingredient for bread fortification. Added edible insects to the bread dough caused increases in the protein and fat content compared to traditional wheat bread. Increased levels of mealworm powder led to a darker appearance and higher total color change. All variants of bread were characterized by good microbiological quality after baking and during 7 days of storage. Overall results indicated that the enrichment level using 5% of mealworm powder in the bread recipe was optimal. Thus, the results confirm the usefulness of *T.molitor* for the protein enrichment of bread. However, further research is needed to investigate the effects of adding edible insects to other food matrices and increase consumer acceptance and awareness.

## Figures and Tables

**Table 1:** Wheat flour and mealworm powder characterization used for bread making.

Physicochemical Properties	Mealworm Powder	Wheat Flour
pH	6.95 ± 0.02 ^b^	6.78 ± 0.13 ^a^
Fat absorption capacity [g oil/g powder]	0.40 ± 0.20 ^a^	0.26 ± 0.05 ^a^
Water holding capacity [g water/g powder]	1.63 ± 0.57 ^a^	1.47 ± 0.14 ^a^
a_w_	0.33 ± 0.00 ^a^	0.44 ± 0.00 ^b^
L*	32.65 ± 1.02 ^a^	80.48 ± 1.34 ^b^
a*	5.84 ± 0.04 ^b^	4.73 ± 0.48 ^a^
b*	25.31 ± 0.21 ^b^	21.54 ± 0.98 ^a^

Values followed by different small letters (a, b) in row are significantly different (*p* ≤ 0.05). Values are means of three replicates ± standard deviation.

**Table 2:** Formulations of doughs obtained with the use of wheat flour (C), wheat and 5% (M5), 10% (M10), 15% (M15) mealworm powder.

	Ingredients [%]					
Samples	Wheat Flour	Mealworm Powder	Freeze-Dried Yeasts	Sugar	Salt	Water
**C**	58.7	0.0	0.8	0.7	0.7	39.2
**M5**	55.8	2.0				
**M10**	52.9	5.9				
**M15**	49.9	8.9				

**Table 3:** Physicochemical properties of obtained breads loaves.

	C	M5	M10	M15
L*	44.87 ± 1.03 ^d^	43.10 ± 0.85 ^c^	41.11 ± 1.45 ^b^	40.07 ± 0.43 ^a^
a*	6.23 ± 0.43 ^b^	6.12 ± 0.12 ^ab^	6.21 ± 0.11 ^b^	6.00 ± 0.26 ^a^
b*	27.93 ± 0.59 ^c^	27.89 ± 0.39 ^c^	27.30 ± 0.18 ^b^	26.92 ± 0.59 ^a^
ΔE	-	2.27 ± 0.12	4.00 ± 0.56	4.50 ± 0.78
BI	13.90 ± 0.14 ^a^	14.26 ± 0.17 ^b^	15.04 ± 0.01 ^c^	14.97 ± 0.33 ^c^
Density [g/cm^3^]	0.43 ± 0.02 ^a^	0.39 ± 0.01 ^a^	0.36 ± 0.00 ^a^	0.36 ± 0.01 ^a^
a_w_	0.97 ± 0.00 ^a^	0.96 ± 0.00 ^a^	0.97 ± 0.00 ^a^	0.97 ± 0.00 ^a^
Hardness [N]	30.91 ± 1.02 ^b^	24.94 ± 0.90 ^b^	28.22 ± 1.56 ^b^	17.12 ± 0.76 ^a^

Values followed by different small letters (a, b, c, d) in row are significantly different (*p* ≤ 0.05). Values are means of three replicates ± standard deviation.

**Table 4:** Composition of bread produced with wheat flour and mealworm powder.

	C	M5	M10	M15
Protein (g)	11.30 ± 0.12 ^a^	14.13 ± 0.11 ^b^	16.95 ± 0.16 ^c^	19.77 ± 0.04 ^d^
Fat (g)	2.23 ± 0.04 ^a^	3.07 ± 0.01 ^b^	3.92 ± 0.02 ^c^	4.77 ± 0.06 ^d^
Carbohydrates (g)	62.09 ± 0.45 ^d^	59.10 ± 1.89 ^c^	56.10 ± 0.49 ^b^	53.11 ± 1.05 ^a^
Fiber (g)	7.47 ± 0.87 ^a^	7.56 ± 0.45 ^a^	7.65 ± 0.61 ^a^	7.74 ± 0.82 ^a^

Values followed by different small letters (a, b, c, d) in row are significantly different (*p* ≤ 0.05). Values are means of three replicates ± standard deviation.

**Table 5:** Microbiological analysis of the tested breads after production and after 2 and 7 days of storage.

Sample	Days	TVC [log CFU g^−1^]	Y&M [log CFU g^−1^]
**C**	0	3.51 ± 0.30 ^a^	nd
	2	7.18 ± 0.07 ^c^	3.58 ± 0.04 ^a^
	7	8.44 ± 0.29 ^e^	4.28 ± 0.03 ^d^
**M5**	0	4.17 ± 0.08 ^b^	nd
	2	6.46 ± 0.44 ^d^	3.34 ± 0.06 ^b^
	7	8.38 ± 0.27 ^e^	4.24 ± 0.05 ^d^
**M10**	0	4.21 ± 0.19 ^b^	nd
	2	6.46 ± 0.45 ^d^	nd
	7	9.17 ± 0.13 ^f^	4.38 ± 0.11 ^d^
**M15**	0	4.22 ± 0.13 ^b^	nd
	2	7.16 ± 0.10 ^c^	3.35 ± 0.04 ^b^
	7	8.58 ± 0.30 ^e^	4.21 ± 0.02 ^d^

The values are expressed as means ± SD, means in the same row followed by different lowercase letters (a–f) are significantly different (*p* < 0.05; TVC—Total Viable Counts; Y&M—Yeasts and Molds; nd—not detected.

**Table 6:** Wheat flour and mealworm powder characterization used for bread making.

	Mealworm Powder	Wheat Flour
Fat (g)	21.5	2.30
Protein (g)	61.0	11.0
Carbohydrates (g)	3.4	63.0
Fiber (g)	6.9	7.60
Energetic value (kcal)	478	332

## Data Availability

There are no data outside that reported in this article.

## Sample Availability

Samples of the compounds are not available from the authors.
